# Correction: Evaluating migration and cytotoxicity of tissue-resident and conventional NK cells in a 3D microphysiological system using live-cell imaging

**DOI:** 10.1039/d5lc90087e

**Published:** 2025-08-11

**Authors:** Hyeri Choi, June Ho Shin, Hyeonsu Jo, John B. Sunwoo, Noo Li Jeon

**Affiliations:** a Interdisciplinary Program in Bioengineering, Seoul National University 1 Gwanak-ro, Gwanak-gu Seoul 08826 Republic of Korea njeon@snu.ac.kr; b Department of Otolaryngology – Head & Neck Surgery, Stanford University 801 Welch Rd Stanford CA 94305 USA sunwoo@stanford.edu; c Department of Mechanical Engineering, Seoul National University 1 Gwanak-ro, Gwanak-gu Seoul 08826 Republic of Korea; d Institute of Advanced Machines and Design (SNU-IAMD), Seoul National University 1 Gwanak-ro, Gwanak-gu Seoul 08826 Republic of Korea

## Abstract

Correction for ‘Evaluating migration and cytotoxicity of tissue-resident and conventional NK cells in a 3D microphysiological system using live-cell imaging’ by Hyeri Choi *et al.*, *Lab Chip*, 2025, **25**, 2696–2707, https://doi.org/10.1039/D4LC01095G.

The authors regret that the spelling of one of the author's names, Noo Li Jeon, was incorrect in the original manuscript. The corrected list of authors for this paper is as shown above.

The Royal Society of Chemistry apologises for these errors and any consequent inconvenience to authors and readers.

